# The Influence of Titania Nanoparticles on the Electrodeposition of Ni-Mo-W Composites in Aqueous Electrolytes at Different Electrolyte Temperatures

**DOI:** 10.3389/fchem.2022.806553

**Published:** 2022-03-11

**Authors:** Usoa Izagirre‐Etxeberria, Elizabeth J. Podlaha

**Affiliations:** ^1^ Department of Chemical Engineering, Northeastern University, Boston, MA, United States; ^2^ TECNALIA, Basque Research and Technology Alliance (BTRA), Parque Tecnológico de San Sebastián, San Sebastian, Spain; ^3^ Department Department of Chemical and Biomolecular Engineering Clarkson University, Potsdam, NY, United States

**Keywords:** electrodeposition, Ni-Mo-W, composite, titania, Hull cell

## Abstract

The electrodeposition of Ni-Mo-W alloys and composites with TiO_2_ are examined with a rotating Hull cell to better understand the influence of the particle on the deposition composition and morphology. The addition of the TiO_2_ particle to the electrolyte and deposit, significantly affected the deposit composition when the electrolyte temperature was 65^0^C. Both Ni and Mo composition in the deposit was enhanced, but not due to higher reaction rates. The enhancement was a result of an apparent inhibition by the hydrogen evolving side reaction. The W partial current density was most significantly inhibited. The deposit morphology changed with the addition of TiO_2_ with a reduction of microcracks compared to the particle-free deposit. The results suggest that the adsorption of the hydrogen intermediate from the side reaction is influenced by the particle, hindering hydrogen desorption, and indirectly affects the partial current densities of the nickel, molybdate and tungstate ion reduction and the morphology.

## Introduction

A challenge in alloy electrodeposition containing Mo, and/or W, is that the deposition exhibits induced codeposition behavior (Brenner, 1963; Landolt, 1994; Schwartz et al., 2004). Induced codeposition is characterized by the inability of molybdenum and tungsten ions in water to be reduced to its zero valence state unlike most transition metal ions. Interestingly, both molybdate and tungstate ions can be coaxed into reduction when codeposited with another element to form an alloy. The elements, which have the best inducing capability, are the iron-group elements: Ni, Co, and Fe. In Brenner’s review (Brenner, 1963) it was recognized that the composition of the solid state alloy was not reflective of the amount of ions in the electrolyte, suggesting a coupled reaction mechanism. Today there is not a general consensus on how these alloys codeposit and thus the control of the deposit composition and subsequent structure leading to the desired properties are still difficult to predict *a priori*. Combined with a second phase particle the deposit composition is even more difficult to predict and work that can contribute to this understanding is needed.

Several comprehensive reviews summarize the induced codeposition mechanism (Fukushima, et al., 1979; Eliaz and Gileadi, 2008; Landolt, 1994; Tsyntsaru et al., 2012; Allahyarzadeh, et al., 2016). In early work by Ernst et al. (1955); (Ernst and Holt, 1958), they suggested that the reduction of Mo or W ions was governed by hydrogen, on account of the observation that generally as more Mo or W is codeposited into an alloy the hydrogen side reaction tends to be higher, or in other words, the current efficiency is lower. Fukushima (Fukushima et al., 1979; Oue, et al., 2009) noted that hydrogen can be readily adsorbed onto the codeposited iron-group element and that the inducing element, such as Mo was reduced at these sites, thus, placing an emphasis on the iron-group solid state as the catalyst in reducing the inducing element (e.g., Mo) and effectively placing a theoretical upper limit for Mo (or W) codeposition in an alloy. Chassaign et al. (1989) reported that the iron-group ion was responsible for the formation of the intermediate, not the solid state. There is also a view that iron-group metal tungstate or molybdate complexes are precursors to tungsten or molybdenum alloys (Younes and Gileadi, 2002; Younes-Metzler et al., 2003) and a contrasting view that the iron-group metal induces adsorption of tungstate or molybdate intermediates at the solid state surface (Podlaha and Landolt, 1996b; Podlaha and Landolt, 1996b; Podlaha and Landolt 1997), and more recent experimental results supported the concept of the adsorbed ion-group element being the governing species to induce the reduction of Mo or W ions according to Eq. 1 through [4] (Sun, et al., 2013), using Ni as one example iron-group element, and where “L” represents a ligand, common in many plating scenarios.

$$MoO_{4}^{2-}+NiL{\left(I\right)}_{ads}+2H_{2}O+2e^{-}\to {\left[Ni\left(I\right)LMoO_{2}\right]}_{ads}^{2-}+4OH^{-}$$ [1]

$${\left[Ni\left(I\right)LMoO_{2}\right]}_{ads}^{2-}+2H_{2}O+4e^{-}↔Mo\left(s\right)+NiL{\left(I\right)}_{ads}+4OH^{-}$$ [2]

and in the same manner for tungstate reduction,

$$WO_{4}^{2-}+NiL{\left(I\right)}_{ads}+2H_{2}O+2e^{-}\to {\left[Ni\left(I\right)LWO_{2}\right]}_{ads}^{2-}+4OH^{-}$$ [3]

$${\left[Ni\left(I\right)LWO_{2}\right]}_{ads}^{2-}+2H_{2}O+4e^{-}↔W\left(s\right)+NiL{\left(I\right)}_{ads}+4OH^{-}$$ [4]

Since the current efficiency can be manipulated to be low or high, by changing the metal ion composition and pH it was thought that the side reaction was not a controlling feature of the induced codeposition mechanism.

Electrodeposited Ni-W and Ni-Mo alloys are of interest for use as corrosion resistant coatings (Quang, et al., 1971; Raman, et al., 2007; Alimadadi et al., 2009), as magnetic materials (Gomez, et al., 2006; Ohgai, et al., 2013), as electrocatalyst for the hydrogen evolution reaction in water splitting (Fan et al., 1994; Navarroi-Flores, et al., 2005; Jamesh, 2016) and as combined wear resistant and corrosion resistant coatings (Urlberger, 1999; Slavcheva et al., 2005; Haseeb et al., 2008). Electrodeposited alloys of Ni-Mo-W may offer combined properties, and has been used as corrosion resistant electrocataysts for the hydrogen evolution reaction (Raj, 1992; Raj and Vasu, 1992; Sun et al., 2013). Cesiulis, et al., 2001 reported enhanced corrosion resistance in amorphous Ni-Mo-W ternary alloy films combined with other interesting properties such as low thermal expansion coefficients and premium hardness. Ni-Mo-W alloys are also important as in creating transition metal sulfides as catalytic precursors on hydrodesulphurization reactions of organic molecules such as tiophene, benzotiophene and dibenzotiophene (Olivas et al., 2009). In this context, the addition of Mo to Ni-W is advantageous as it promotes an amorphous structure when treated at high temperature with H_2_S/H_2_ and results in a Ni-Mo-W-S active catalyst. Composites of Ni-W and Ni-Mo can permit further tailorability of properties. For example, electrodeposited Ni-Mo-ZrO_2_ coatings have been reported to improve microhardness and corrosion properties of Ni-Mo alloy coatings (Laszczynska, et al., 2016) and electrodeposited Ni-W-SiC composites have been reported to enhance the corrosion resistance, hardness and wear resistance over that of Ni-W coatings (Yao et al., 2017; Singh, et al., 2016), as SiC it is known to improve properties in Ni films (Lee, et al., 2007)*.* Titania particles have been codeposited with Ni-W coatings and both the hardness and corrosion resistance was improved by the presence of titania (Kumar, et al., 2013). Enhanced electrocatalysis of the hydrogen evolution reaction (HER) in water splitting application has been reported for Ni-W-TiO_2_ composite in comparison to Ni-W due to a rougher surface that was created by the addition of the TiO_2_ particle (Zou, et al., 2004), associated with a change in the morphology. Zhang et al. (2018) have reported an intrinsic enhancement of HER kinetics for Ni-W-TiO_2_ nanocomposite coatings compared to the nanoparticle-free counterparts. Few studies address the influence of the particle on the alloy composition that can have a large impact of the deposit composition. Previous work with Ni-W-TiO_2_ showed that the deposit tungsten content was slightly decreased with accompanying titania (Zhang and Podlaha-Murphy, 2017). Here, electrodeposited Ni-Mo-W and Ni-Mo-W-TiO_2_ composites are examined with a focus on addressing the role of the particle on the metal reduction rates (*i.e*., partial current densities) that dictate the composition.

A convenient way to assess deposit composition is with the trapezoidal Hull cell (Hull, 1939) that generates a current distribution along the working electrode surface created by cell geometric considerations, and when the current distribution is categorized as a primary current distribution, i.e., under conditions when kinetics are rapid and ohmic effects are dominate. Nobe adapted this technique to alloy electrodeposition (e.g., Wei, et al., 2008) to swiftly identify the composition and morphology of the deposit with variable current density. A rotating version of the Hull cell (RHC) provides better control of the hydrodynamic environment at the cathode surface via control of the rotation rate. Madore and Landolt, 1993 presented design conditions for obtaining a large variation in current distribution along the length of a cathode for the Ni-Cu system that mimics the distribution that is created in a conventional Hull cell. The current distribution is generated by placing the anode at either bottom or top of a rotating cylinder that is shield by a plastic tube, and open only at one end. If the anode is placed near the bottom of the cell and the insulating tube is open at the bottom then the current density is highest in this region and decreases along the cathode cylinder length. They demonstrated the deconvolution of partial current densities in a Ni-Cu alloy by mapping the current distribution to a polarization curve. The work presented here will follow this established approach and is the first demonstration of the use of the RCHC of the electrodeposition of Ni-W-Mo-TiO_2_ composites.

## Experimental

Two electrode experimental set-ups were used: *i*. a rotating cylinder electrode (RCE) with uniform current distribution. and *ii*. a rotating cylindrical Hull cell (RCHC), with non-uniform distribution, as the working electrode. In both cases copper was the substrate. In the RCE configuration, copper cylindrical electrodes with a diameter of 0.6 and 1.0 cm length were used. In the RCHC configuration, the copper cylindrical rods were longer, having a length of 8 cm length. The counter electrode was a platinum coated, titanium anode. The electrolyte contained 0.15 M nickel sulfate, 0.005 M sodium molybdate, 0.375 M sodium citrate, 0.1 M sodium tungstate and 0.1 M boric acid. Different electrolyte temperatures were examined: 25°C, 45°C and 65°C. The electrolyte pH was maintained at 7 with sodium hydroxide and sulfuric acid additions. The TiO_2_ particle type added to the electrolyte were an anatase nanopowder, with purity of 99.7% supplied by Sigma Aldrich, with a reported diameter size lower than 25 nm. A low particle loading of 12.5 g L^−1^ was used so as not to significantly influence the hydrodynamics of the RCE and RCHC electrodes.

The RCHC experiments were obtained using an average current density of 66.3 mA cm^−2^ using a Solartron SI 1287 galvanostat for a period of 25 min at a constant rotation rate of 500 rpm. The rotation rate was controlled with a Pine Instruments modulated speed high precision rotator. The temperature was controlled using a thermostatic bath. An estimate of the local current density, *i* (z)*,* along the dimensionless position, *z*, was determined from a primary current distribution correlation described by the following (Madore and Landolt, 1993),

$$\frac{i\left(z\right)}{i_{avg}\;}=\;\frac{0.535-0.458\;z}{{\left[0.0233+{\left(z\right)}^{2}\right]}^{1/2}}+8.52\times \;10^{-5}\exp\left\{7.17\left(z\right)\right\}$$ [5]

where i_avg_ is the average applied current density. Hence the variation of the current density along the cathode length can be estimated to provide a rapid evaluation of the deposit composition and morphology as a function of current density.

A computer controlled potentiostat and impedance system (Solartron SI 1287 potentiostat coupled to Solartron Analytical 1252A frequency Response Analyzer) were used to conduct the potentiodynamic scans. Polarization curves were measured with and without titania nanoparticles on a RCE electrode at the three examined temperatures and at a constant rotation rate of 500 rpm. A saturated calomel electrode was used as the reference electrode. The polarization scans were performed by increasing the cell potential from the open circuit value until -3.0 V vs SCE and corrected for ohmic drop with impedance spectroscopy.

An X-Ray fluorescence (XRF) instrument, model Kevex Omicron, was used to analyze composition and thickness of the deposits. Thirty points were analyzed along each of the cylindrical cathodes. The TiO_2_ amount of the deposits was confirmed using a scanning electron microscope, model Hitachi S4800 field emission coupled to an EDAX detecting unit, which provides an order of magnitude higher precision. The thickness was confirmed gravimetrically. The partial current densities of Ni, Mo and W were calculated from the thickness and composition analysis using Faraday´s law. Scanning electron microscopy was also used to examine the morphology of characteristic deposits.

## Results

**FIGURE 1:**
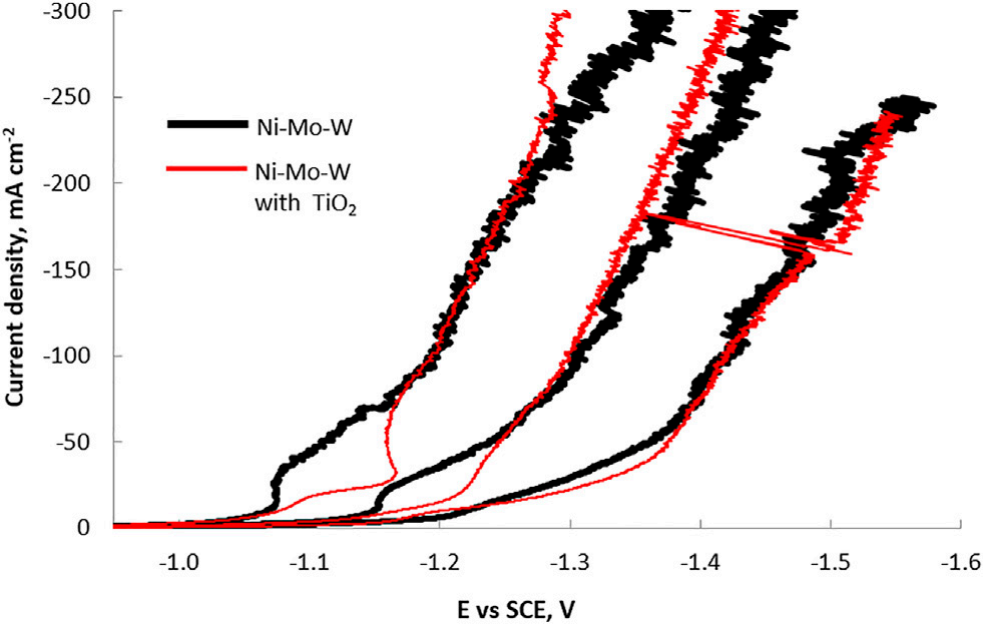
RCE polarization curves from the electrodeposition of a Ni-Mo-W alloy for a particle free electrolyte and in the presence of titania particles at 25°C, 45°C and 65°C.

The polarization curves in Figure 1 show the effect of the addition of TiO_2_ particles on the total current density during Ni-Mo-W ternary electrodeposition at different electrolyte temperatures and at a rotation rate of 500 rpm. The total current density is inhibited when titania nanoparticles are present in the electrolyte at low current densities with the degree of change largest at 65°C and lowest at 25°C. The inhibition of the total current density increases notably with temperature. At more negative potential values than -1.3 V, -1.2 V and -1.0 V vs SCE, at 25°C, 45°C and 65°C, respectively, the total current density is not notably inhibited nor enhanced by the addition of the nanoparticles. In order to analyze which reaction causes the changes in the total current density observed in the different regions, the partial current densities were examined with the RCHC, with a forced variation in current distribution along the cathode length.

**FIGURE 2:**
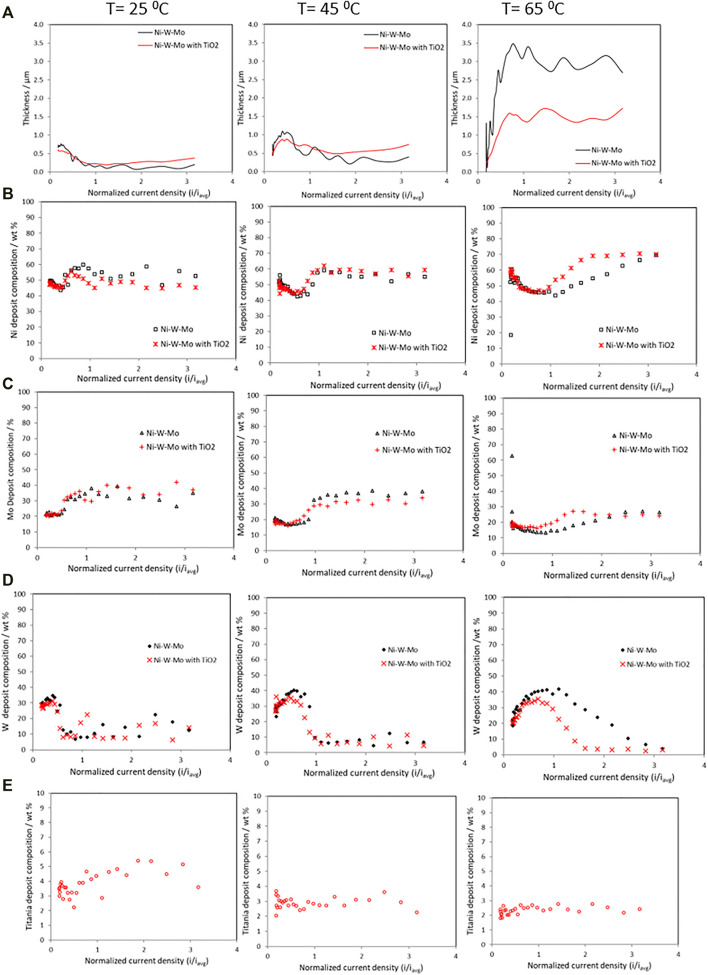
Deposit composition measurements on a rotating Hull cell during the electrodeposition of Ni-Mo-W with and without TiO_2_, at 25°C, 45°C and 65°C, **(A)** thickness variation **(B)** Ni wt%, **(C)** Mo wt%, **(D)** W wt% and **(E)** TiO_2_ wt%.

The thickness and the composition change of the composite along the RCHC electrode length, at 25°C, 45°C and 65°C, is shown in Figure 2. The deposit thickness (Figure 2A) at 25°C and 45°C, did not change appreciably with the addition of the TiO_2_ particles. However, at the higher temperature, 65°C, the addition of titania particles significantly lowered the deposit thickness compared to the alloy without nanoparticles. Generally, at the high temperature of 65°C, there is a much thicker deposit compared to the lower temperatures, even with the addition of particles. The composition of nickel, (Figure 2B), molybdenum (Figure 2C), and tungsten (Figure 2D) with and without the addition of 12.5 g L^−1^ of TiO_2_ particles, show that there is a similar change in the Ni and Mo composition with current density, with a drop in composition as the current density increases and then a rise in its composition, with the tungsten exhibiting the opposite behavior. There is slightly more Ni in the deposit at the low and high current density regions at 65°C compared to 25°C and 45°C, when there is no TiO_2_ present in the electrolyte, however when *i*/*i*_*avg*_ has a value of 1–2 (66.3–132.6 mA cm^−2^), the amount of Ni in the deposit is larger at the lower temperatures. The addition of the TiO_2_ particles only has a significant effect on the Ni deposit composition at the high temperature and at high current density. At the low current density region, there is a comparable amount of W and Mo despite having 20 times more tungstate in the electrolyte than molybdate. With a change of temperature, the composition does change when there are no particles present depending on the applied current density. In the low current density region, there is a drop in the Mo deposit content with an increase in temperature, but at 25°C, the composition reaches a peak at 38 wt% but then falls with an increase in the applied current density, while at 45°C, at high current density, the composition remains near the same 38 wt% so is higher than when the electrolyte temperature is at 25°C. A further increase in the temperature to 65°C leads to lower Mo content at all current densities. There is little difference in Mo composition when TiO_2_ particles are present or not at the low current density region for all temperatures. In the medium range of current density the addition of particles does not significantly affect the Mo deposition composition at 25°C and at 45°C, but slightly increases it at 65°C. The behavior of the W composition with current density rises with the applied current density with or without particles, reaches a maximum, and then falls to nearly zero, with an associated rise in Mo and Ni. A similar drop in the tungsten amount with an increase of applied current density, has been previously reported (Sun et al., 2013) in a Ni-Mo-W electrodeposit when no TiO_2_ particles are in the electrolyte; here the particles exaggerate this effect. The enhancement of the Ni composition with the particle addition was observed in the medium and high current density regions while the enhancement of molybdenum was observed in the low and medium current density regions. The TiO_2_ particle concentration in the resulting Ni-W-Mo-TiO_2_ composite thin films is shown in Figure 2E. The titania amount incorporated into the composite coatings (Figure 2E) decreased as temperature increased from 25°C to 65°C.

**FIGURE 3:**
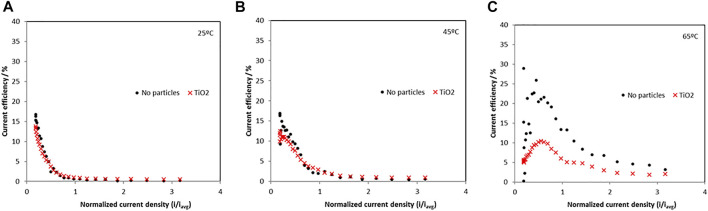
Current efficiency of Ni-Mo-W electrodeposition with and without addition of titania nanoparticles at **(A)** 25°C, **(B)** 45°C and **(C)** 65°C.

The current efficiency at 25°C, 45°C and 65°C is shown in Figure 3 and are relatively low indicating a significant hydrogen evolution side reaction. When no particles are present, the maximum current efficiencies occurred in the low current density regions and dropped as current was increased. The maximum current efficiency increased as temperature increased from 25°C to 65°C. At 65°C the current efficiency had a maximum of 25% in the low-density region without particles and decreased to a maximum of around 10% with the addition of particles.

**FIGURE 4:**
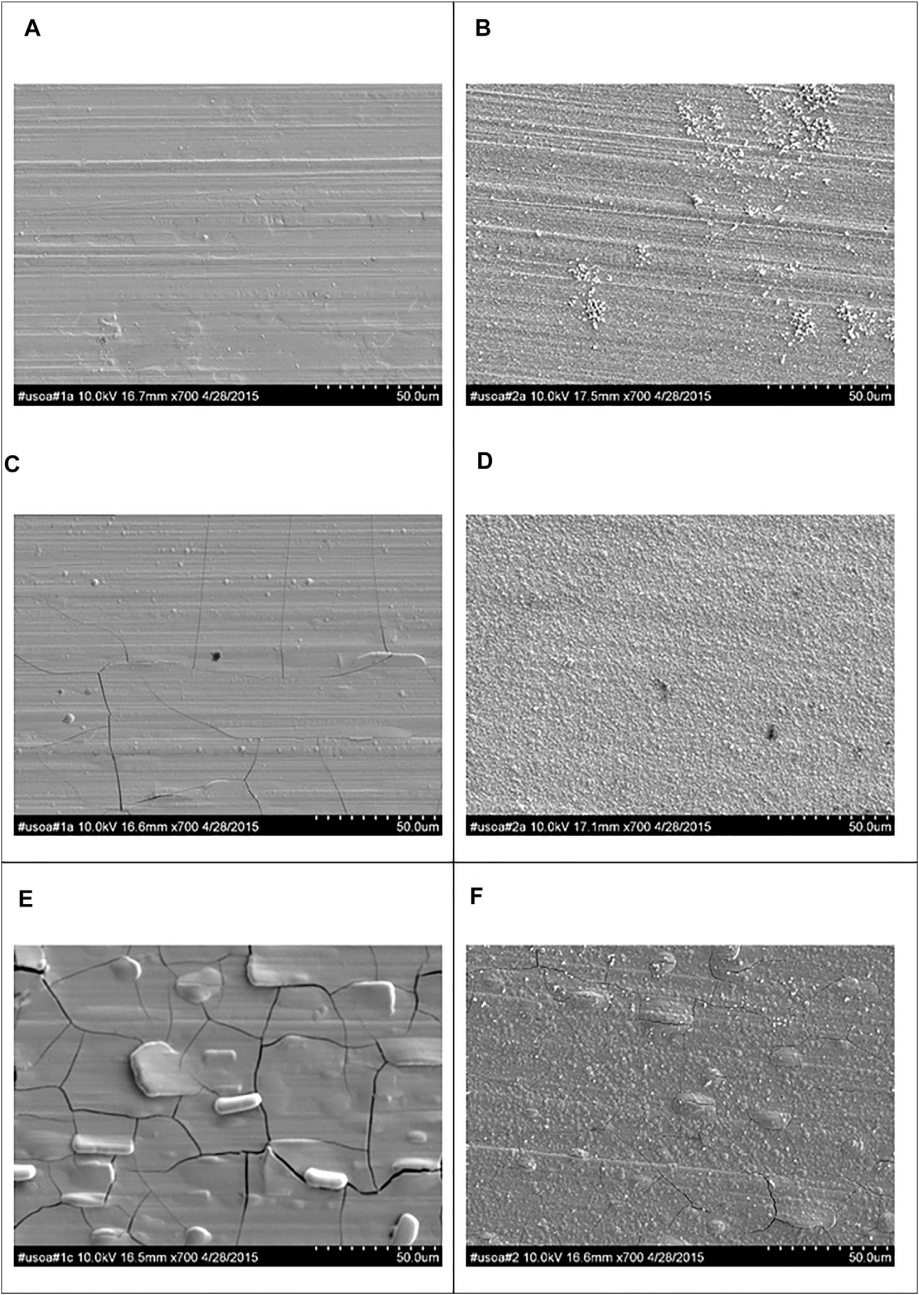
SEM micrographs at the **(A,B)** low **(C,D)** medium and **(E,F)** high current density regions of RHC samples electrodeposited during 25 min at 65°C and 500 rpm (a,c,e) without and (b,d,f) with the addition of 12.5 g L^−1^ of titania nanoparticles.

Figure 4 shows representative SEM micrographs at three regions on the RCHC electrodeposit at low, medium and high current densities at 65°C and from the electrolytes with and without the addition of TiO_2_ nanoparticles. The Ni-W-Mo alloy deposit (Figure 4A,C,D) showed a smooth surface morphology with a progressive increase of micro-cracks as the current density increased. When particles were added, at the same current density regions, (Figure 4B,E,F) significantly fewer micro-scale cracks were observed but the deposit was more nodular.

## Discussion

**FIGURE 5:**
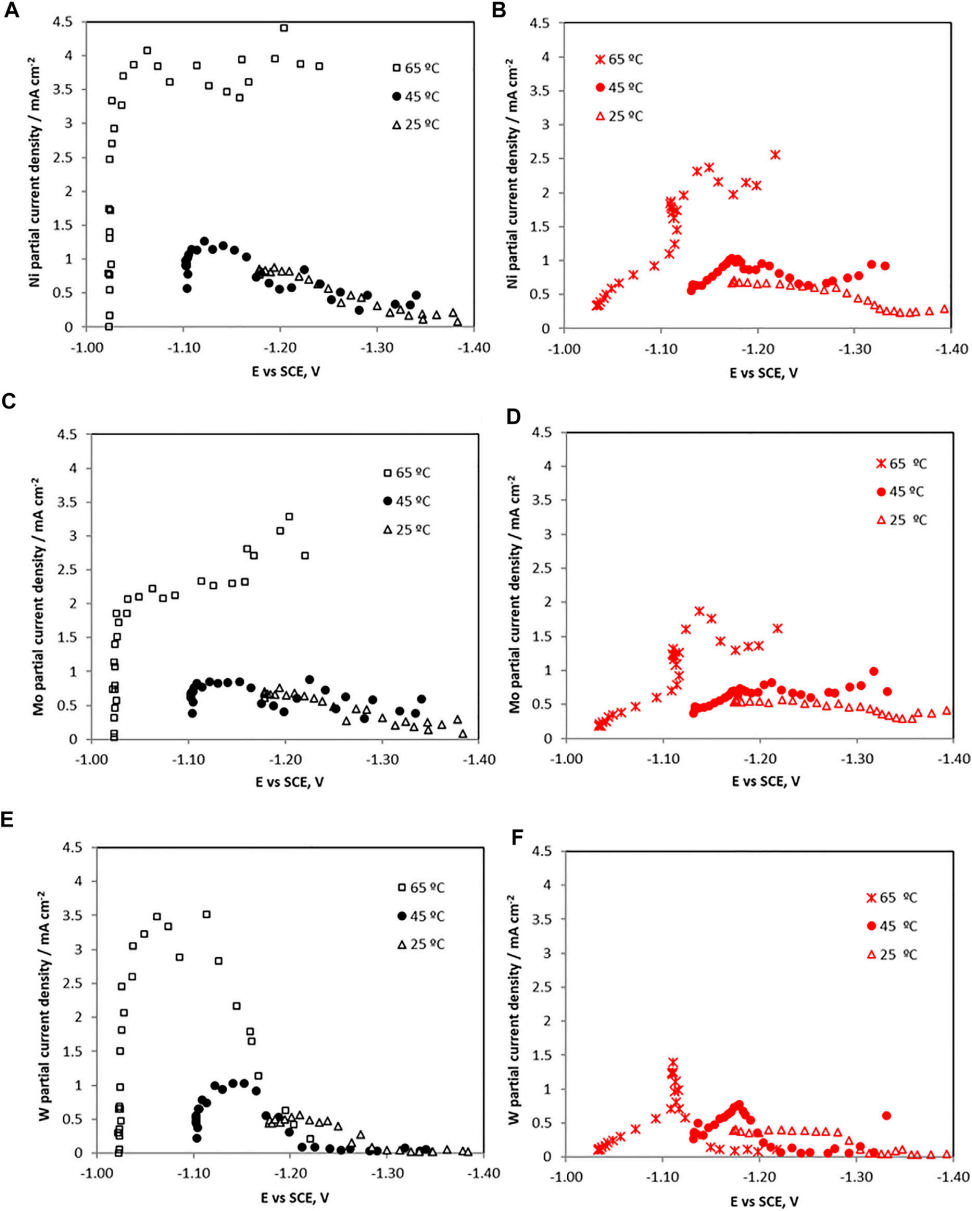
The partial current densities with potential of each metal ion reduction without (left) and with (right) TiO_2_ particles for **(A,B)** Ni **(C,D)** Mo, and **(E,F)** W during the electrodeposition of Ni-Mo-W at different electrolyte temperatures.

**FIGURE 6:**
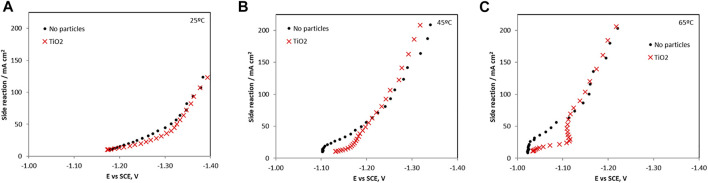
The hydrogen evolution side reaction partial current densities with and without the addtion of TiO2 in the electrolyte **(A)** 25°C, **(B)** 45°C, and **(C)** 65°C.

The partial current densities of nickel, molybdenum, tungsten and the side reaction were determined to provide further insight on how the reaction rates were affected by temperature and the particle addition. The partial current densities of the metal ion reduction rates and the side reaction rate is shown in Figures 5, 6, respectively, determined using Faraday’s law from the composition and thickness along the RCHC length. Using the polarization curves in Figure 1, the *x*-axis scale was correlated with the applied working electrode potential.

An increase in the temperature shifts the metal reduction rates to less positive potentials (*i.e*., depolarizes the deposition). The side reaction is also depolarized with temperature. When no TiO_2_ particles are present, all the partial current densities of the individual metals increase with potential, when the total current density is confined to the low region and the side reaction is small. This potential dependence indicates a kinetic reaction control. The Ni and Mo partial current densities exhibit a sharp change and then remain relatively constant at 65°C, between −1 and −1.2 V vs SCE. At lower temperatures the Ni and Mo partial current densities decrease in this potential range. Only the tungsten partial current density suffers a drop at higher overpotentials, occurring when the side reaction, associated with a large increase in the slope of the hydrogen evolution reaction. To determine if the horizontal region of the Ni and Mo partial current densities at 65°C are reflective of a mass transport control region, the limiting current densities were calculated using the empirical Eisenberg equation (Eisenberg, et al., 1954).

$$i_{L}=0.01nFC^{b}D^{0.644}v^{-0.344}S^{0.7}d_{i}^{0.4}$$ [6]

where *n* is the number of electrons transferred, *F* is Faraday´s constant, *C* ^*b*^ is the metal bulk concentration, and *D* is the metal diffusion coefficient. Assuming diffusion coefficients of nickel, molybdenum and tungsten to be close to 5 x 10^–6^ cm^2^ s^−1^, electrons transferred of 2, 6 and 6 for nickel, molybdenum and tungsten, respectively, with an estimated kinematic viscosity, *v*, using that of water, 0.01 cm^2^ s^−1^, an electrode rotation rate, S, of 500 rpm and cathode diameter, *d*_*i*_ 0.6 cm, the resulting limiting current density values for the individual metal ions reduction reactions are 34, 3, and 69 mA cm^−2^ for Ni, Mo and W, respectively. The calculated limiting current density value for molybdenun presents the same order of magnitude than that observed in Figure 5, so the molybdenum electrodeposition may be under a mass transport control at the higher total current density region, attributable to the low concentration used. In the case of nickel and tungsten, the calculated limiting current densities were one order of magnitude higher than those observed. Hence, these metals did not reach their mass transport limited current densities so kinetic effects control their deposition, even when the Ni partial current density appears horizontal.

When TiO_2_ particles were present, all the metal ion reduction reaction rates (*i.e*., partial current densities) are shifted to more negative, less noble potentials, and inhibited at 65°C. At the lower temperatures the metal reduction rates were not significantly inhibited with the addition of the TiO_2_. Interestingly, the side reaction rate (Figure 6) is progressively more inhibited with temperature at the low current density region. At 65°C, it is the most inhibited with added particles until −1.12 V vs SCE. At this point, the side reaction rate increases sharply with an associated inflection in the nickel and molybdenum partial current densities. The tungsten current density exhibited a maximum at -1.12 V vs SCE and dropped to zero at higher overpotentials. For the side reaction, its partial current density reached the values measured in the electrolyte without particles at potential values more negative than −1.12 V. Thus, the decrease in the composite efficiency (Figure 3) at high current density was attributed primarily with a decrease of the metal rates, not an increase in the side reaction. In the region between 1.1 V and −1.2 V vs SCE the total current density in Figure 1 decreased when particles were present due to the lowering of all the reaction rates, both the metal and the side reaction.

Correlating these features with the composition of the Ni-Mo-W alloy material electrodeposited at 65°C, (Figure 2) the maximum tungsten composition (∼40 wt%) was achieved at the lower overpotential values, i.e. when the tungsten partial current density was maximum. Then, at higher overpotentials, the deposit continued to be enriched in nickel and in molybdenum mainly due to the drop of the tungsten partial current density, since nickel and molybdenum partial current densities remained practically constant with potential. When the particles were present, interestingly, there is a current density region where the nickel and molybdenum composition in the deposit increases, with an associated decrease in tungsten wt%. This behavior is not attributed to an enhancement of the nickel and molybdenum partial current densities but in fact a decrease, with less of a relative decrease compare to tungsten partial current density.

According to the Sun-Bairchanya-Podlaha model (Sun, et al., 2013), the observed lowering of the partial current densities is consistent with a decrease of the adsorbed mixed-metal intermediates, shown in equations (1)–(4). Since there is a substantial inhibition of the tungsten at larger overpotentials, or larger applied cathodic current densities, there may be an associated decrease in the $\mathrm{formation}\;\mathrm{of}\;\mathrm{the}\;{\left[Ni\left(I\right)LWO_{2}\right]}_{ads}^{2-}$ intermediate. The large inhibition occurs at a point when the side reaction increases (compare Figure 5 and Figure 6 (c)) and that may be due to the added adsorbed hydrogen species.

The hydrogen evolution reaction on Ni-W electrodeposits is known to follow a Volmer-Heyrovsky mechanism in alkaline electrolytes (Popczyk and Losiewicz, 2015),

$$H_{2}O+M+e^{-\;}\to M-H_{ads}+OH^{-\;}$$ [7]

$$H_{2}O+M-H_{ads}+e^{-\;}\to H_{2}+M+OH^{-\;}$$ [8]

with expected adsorption of hydrogen, *H*_*ads*_, at the electrode surface. Since the drop of the tungsten partial current density coincides with the large increase in the hydrogen evolution side reaction, it may be that this adsorbed intermediate is complicit in interfering with the tungsten reduction. However, the nickel and molybdenum are affected to a lesser extent by the adsorbed hydrogen. The adsorption of all species are limited by the available surface area. The impact of the *H*_*ads*_ has less an effect on Ni and Mo, suggesting that there is a smaller change of the adsorbed intermediates of *Ni(I)*_*ads*_ and ${\left[Ni\left(I\right)LMO_{2}\right]}_{ads}^{2-}$ . Adsorption energies are temperature dependent and thus changes in temperature are expected to alter the fractional coverage of the intermediates. Thus it is possible that there is a composition for surface sites where *H*_*ads*_ *, Ni(I)*_*ads*_ and ${\left[Ni\left(I\right)LMO_{2}\right]}_{ads}^{2-}$ is favored over ${\left[Ni\left(I\right)LWO_{2}\right]}_{ads}^{2-}$ .

The small amount of particles in the deposit and in the electrolyte has a substantial impact on the reaction rates, lowering them, including the side reaction, considerably. Since the current efficiency is reduced (Figure 3) when the particles are added, the relative amount of the total metal reduction rates is reduced more than the hydrogen evolution side reaction at the intermediate current densities. At high applied current densities, or larger overpotential, the side reaction occurring when the particles are present is not altered, but the metal rates are reduced leading to an even larger decrease in the current efficiency. Thus, the particles may be indirectly affecting the *H*_*ads*_, which then impacts all the other adsorbed species.

The particle also lowers the extent of cracking of the deposit. Since cracks may be due to the absorbed hydrogen as noted in Co-Mo (Gómes et al., 2001) and Ni-W electrodeposits (Younes and Gileadi, 2000) the particle may be decreasing the absorbed hydrogen and hence enhancing the adsorbed H_ads_ intermediate on the surface. Thus, a model consistent with the partial current density observations and the changes in cracking is that the TiO_2_ particles changes the surface environment to promote the hydrogen intermediate adsorption $H_{ads}$ . This intermediate has a larger effect to poison the tungstate ion reduction compared to both nickel and molybdate ion reduction, suggesting that the adsorption intermediates of nickel and molybdate is considerably greater than that of tungsten.

From a practical point of view, the electrodeposition of Ni-W-Mo alloy coatings reinforced with TiO_2_ nanoparticles can be a good strategy to combine corrosion resistant coatings with electrocatalysis, for applications such as water splitting, since it has been recently recognized that the addition of TiO_2_ can also improve the hydrogen evolution reaction in basic electrolytes in Mo-alloys (Zhang, et al., 2018; Wang et al., 2019, Wang et al., 2020). The addition of TiO_2_ also is beneficial to reduce the extent of cracks and to provide improved roughness to the surface, important to surface finishing applications. However, electrodeposition parameters such as current density must be very carefully controlled since TiO_2_ incorporation can be detrimental to incorporate tungsten that contributes to the overall corrosion and wear resistance.

## Conclusion

Ni-Mo-W and Ni-Mo-W-TiO_2_ composites were electrodeposited from aqueous citrate electrolytes with an increase in the electrolyte temperature resulting in a positive potential shift of the deposition and higher current efficiency. It is the first report of Ni-Mo-W-TiO_2_ electrodeposits. However, at a higher electrolyte temperature (65^0^C), the deposit composition was largely influenced by the addition of TiO_2_ particularly at high current densities that coincided with a large rise in the hydrogen evolution side reaction. The addition of TiO_2_ did not promote any metal reduction reaction. Changes in the deposit composition were due to different extents of metal partial current density inhibition, thought to be due to an increase in adsorbed hydrogen from the side reaction.

## Data Availability

The raw data supporting the conclusions of this article will be made available by the authors, without undue reservation.

## References

[B1] Alimadadi H. AhmadiAliofkhazraei M. M. Aliofkhazraei M. Younesi S. R. (2009). Corrosion Properties of Electrodeposited Nanocrystalline and Amorphous Patterned Ni-W alloy. Mater. Des. 30, 1356–1361. 10.1016/j.matdes.2008.06.036

[B2] Allahyarzadeh M. H. Aliofkhazraei M. Rezvanian A. R. Torabinejad V. Sabour Rouhaghdam A. R. (2016). Ni-W Electrodeposited Coatings: Characterization, Properties and Applications. Surf. Coat. Technol. 307, 978–1010. 10.1016/j.surfcoat.2016.09.052

[B3] Arulraj I. (1992). Nickel Based Composite Electrolytic Surface Coatings as Electrocatalysts for the Cathodes in the Energy Efficient Industrial Production of Hydrogen from Alkaline Water Electrolytic Cells. Int. J. Hydrogen Energ. 17, 413–421. 10.1016/0360-3199(92)90185-y

[B4] Arunsunai Kumar K. Paruthimal Kalaignan G. Muralidharan V. S. (2013). Direct and Pulse Current Electrodeposition of Ni-W-TiO2 Nanocomposite Coatings. Ceramics Int. 39, 2827–2834. 10.1016/j.ceramint.2012.09.054

[B5] Brenner A. (1963). Electrodeposition of Alloys: Principles and Practice. New York: Academic Press, 399–450.

[B6] Cesiulis H. Baltutiene A. Donten M. Donten M. Stojek Z. (2001). Increase in Rate of Electrodeposition and in Ni(II) Concentration in the bath as a Way to Control Grain Size of Amorphous/nanocrystalline Ni-W Alloys. J. Solid State. Electrochem. 6, 237–244. 10.1007/s100080100225

[B7] Chassaing E. Vu Quang K. Wiart R. (1989). Mechanism of Nickel-Molybdenum alloy Electrodeposition in Citrate Electrolytes. J. Appl. Electrochem. 19, 839–844. 10.1007/bf01007931

[B8] Eisenberg M. Tobias C. W. Wilke C. R. (1954). Ionic Mass Transfer and Concentration Polarization at Rotating Electrodes. J. Electrochem. Soc. 101, 306–319. 10.1149/1.2781252

[B9] Eliaz N. Gileadi E. (2008). “Induced Codeposition of Alloys of Tungsten, Molybdenum and Rhenium with Transition Metals, in Modern Aspects of Electrochemisry,” in Modern Aspects of Electrochemistry. Editors Vayenas C. G. White R. E. Gamboa-Aldeco M. E. (New York, NY: Springer), Vol. 42.

[B10] Ernst D. W. Amlie R. F. Holt M. L. J. (1995). Electrodeposition of Molydenum Alloys from Aqueous Solutions. J. Electrochem. Soc. 102, 461–469.

[B11] Ernst D. W. Holt M. L. (1958). Cathode Potentials during the Electrodeposition of Molybdenum Alloys from Aqueous Solutions. J. Electrochem. Soc. 105, 686–692. 10.1149/1.2428691

[B12] Fan C. Piron D. L. Sleb A. Paradis P. (1994). Study of Electrodeposited Nickel‐Molybdenum, Nickel‐Tungsten, Cobalt‐Molybdenum, and Cobalt‐Tungsten as Hydrogen Electrodes in Alkaline Water Electrolysis. J. Electrochem. Soc. 141, 382–387. 10.1149/1.2054736

[B13] Fukushima H. Akiyama T. Akagi S. Higashi K. (1979). Role of Iron-Group Metals in the Induced Codeposition of Molybdenum from Aqueous Solution. Trans. JIM 20, 358–364. 10.2320/matertrans1960.20.358

[B14] Gómes E. Pellicer E. Vallés E. (2001). Electrodeposited Cobalt-Molybdenum Magnetic Materials. J. Electroanalytical Chem. 517, 109–116. 10.1016/S0022-0728(01)00682-9

[B15] Gómez E. Pellicer E. Duch M. Esteve J. Valles E. (2006). Molybdenum alloy Electrodeposits for Magnetic Actuation. Electrochimica Acta 51, 3214–3222. 10.1016/j.electacta.2005.09.010

[B16] Haseeb A. S. M. A. Albers U. Bade K. (2008). Friction and Wear Characteristics of Electrodeposited Nanocrystalline Nickel-Tungsten alloy Films. Wear 264, 106–112. 10.1016/j.wear.2007.02.004

[B17] Hull R. O. (1939). Apparatus and Process for the Study of Plating Solutions. US patent No. 2,149,344.

[B18] Jamesh M. I. (2016). Recent Progress on Earth Abundant Hydrogen Evolution Reaction and Oxygen Evolution Reaction Bifunctional Electrocatalyst for Overall Water Splitting in Alkaline media. J. Power Sourc. 333, 213–236. 10.1016/j.jpowsour.2016.09.161

[B19] Landolt D. (1994). Electrochemical and Materials Science Aspects of alloy Deposition. Electrochimica Acta 39, 1075–1090. 10.1016/0013-4686(94)e0022-r

[B20] Laszczynska A. Winiarski J. Szczygiel B. Szczygiel I. (2016). Electrodeposition and Characterization of Ni-Mo-ZrO_2_ Composite Coatings. Appl. Surf. Sci. 369, 224–231. 10.1016/j.apsusc.2016.02.086

[B21] Lee H.-K. Lee H.-Y. Jeon J.-M. (2007). Codeposition of Micro- and Nano-Sized SiC Particles in the Nickel Matrix Composite Coatings Obtained by Electroplating. Surf. Coat. Technol. 201, 4711–4717. 10.1016/j.surfcoat.2006.10.004

[B22] Madore C. Landolt D. (1993). The Rotating Cylinder Hull Cell - Design and Application. Plating Surf. Finishing 80, 73–78.

[B23] Navarro-Flores E. Chong Z. Omanovic S. (2005). Characterization of Ni, NiMo, NiW and NiFe Electroactive Coatings as Electrocatalysts for Hydrogen Evolution in an Acidic Medium. J. Mol. Catal. A: Chem. 226, 179–197. 10.1016/j.molcata.2004.10.029

[B24] Ohgai T. Tanaka Y. Washio R. (2013). Nanocrystalline Structure and Soft Magnetic Properties of Nickel-Molybdenum alloy Thin Films Electrodeposited from Acidic and Alkaline Aqueous Solutions. J. Solid State. Electrochem. 17, 743–750. 10.1007/s10008-012-1924-z

[B25] Olivas A. Galván D. H. Alonso G. Fuentes S. (2009). Trimetallic NiMoW Unsupported Catalysts for HDS. Appl. Catal. A: Gen. 352, 10–16. 10.1016/j.apcata.2008.09.022

[B26] Oue S. Nakano H. Kobayashi S. Fukushima H. (2009). Structure and Codeposition Behavior of Ni-W Alloys Electrodeposited from Ammoniacal Citrate Solutions. J. Electrochem. Soc. 156, D17–D22. 10.1149/1.3006389

[B27] Podlaha E. J. Landolt D. (1996a). Induced Codeposition: I. An Experimental Investigation of Ni‐Mo Alloys. J. Electrochem. Soc. 143, 885–892. 10.1149/1.1836553

[B28] Podlaha E. J. Landolt D. (1996b). Induced Codeposition: II. A Mathematical Model Describing the Electrodeposition of Ni‐Mo Alloys. J. Electrochem. Soc. 143 (3), 893–899. 10.1149/1.1836554

[B29] Podlaha E. J. Landolt D. (1997). Induced Codeposition: III. Molybdenum Alloys with Nickel, Cobalt, and Iron. J. Electrochem. Soc. 144, 1672–1680. 10.1149/1.1837658

[B30] Popczyk M. Łosiewicz B. (2015). Influence of Surface Development of Ni/W Coatings on the Kinetics of the Electrolytic Hydrogen Evolution. Ssp 228, 293–298. 10.4028/www.scientific.net/ssp.228.293

[B31] Quang K. V. Chassaing E. Bourelier F. Montuelle J. (1971). Uncracked Electrolytic Nickel-Molybdenum alloy Platings and Heat Treatments for High Resistance to Acid. Corrosion 19, 237–248.

[B32] Raj I. A. Vasu K. I. (1992). Transition Metal-Based Cathodes for Hydrogen Evolution in Alkaline Solution: Electrocatalysis on Nickel-Based Ternary Electrolytic Codeposits. J. Appl. Electrochem. 22, 471–477. 10.1007/bf01077551

[B33] Raman K. R. S. G. S. Raman S. G. S. Seshadri S. K. (2007). Corrosion Behaviour of Electrodeposited Nanocrystalline Ni-W and Ni-Fe-W Alloys. Mater. Sci. Eng. A 460-461, 39–45. 10.1016/j.msea.2007.02.055

[B34] Schwartz M. Myung N. V. Nobe K. (2004). Electrodeposition of Iron Group-Rare Earth Alloys from Aqueous Media. J. Electrochem. Soc. 151, C468–C477. 10.1149/1.1751196

[B35] Singh S. Sribalaji M. Wasekar N. P. Joshi S. Sundararajan G. Singh R. (2016). Microstructural, Phase Evolution and Corrosion Properties of Silicon Carbide Reinforced Pulse Electrodeposited Nickel-Tungsten Composite Coatings. Appl. Surf. Sci. 364, 264–272. 10.1016/j.apsusc.2015.12.179

[B36] Slavcheva E. Mokwa W. Schnakenberg U. (2005). Electrodeposition and Properties of NiW Films for MEMS Application. Electrochimica Acta 50, 5573–5580. 10.1016/j.electacta.2005.03.059

[B37] Sun S. Bairachna T. Podlaha E. J. (2013). Induced Codeposition Behavior of Electrodeposited NiMoW Alloys. J. Electrochem. Soc. 160, D434–D440. 10.1149/2.014310jes

[B38] Tsyntsaru N. Cesiulis H. Donten M. Sort J. Pellicer E. Podlaha-Murphy E. J. (2012). Modern Trends in Tungsten Alloys Electrodeposition with Iron Group Metals. Surf. Engin. Appl.Electrochem. 48, 491–520. 10.3103/s1068375512060038

[B39] Urlberger H. H. (1999). Coatings with High Efficiency. Corrosion- and Wear -resistant Nickel Alloys. Metalloberflaeche 53, 15–18.

[B40] Wang C. Bilan H. K. Podlaha E. J. (2019). Electrodeposited Co-mo-TiO2 Electrocatalysts for the Hydrogen Evolution Reaction. J. Electrochem. Soc. 166, F661–F669. 10.1149/2.1091910jes

[B41] Wang C. Podlaha E. J. (2020). Communication-Electrodeposited Co-mo-P-TiO2 Composites Electrocatalysts for the Hydrogen Evolution Reaction. J. Electrochem. Soc. 167, 132502–132506. 10.1149/1945-7111/abb7e7

[B42] Wei J. C. Schwartz M. Nobe K. (2008). Aqueous Electrodeposition of SmCo Alloys. J. Electrochem. Soc. 155, D660–D665. 10.1149/1.2961013

[B43] Yao Y. Yao S. Zhang L. Wang H. (2007). Electrodeposition and Mechanical and Corrosion Resistance Properties of Ni-W/SiC Nanocomposite Coatings. Mater. Lett. 61, 67–70. 10.1016/j.matlet.2006.04.007

[B44] Younes O. Gileadi E. (2000). Electroplating of High Tungsten Content Ni/W Alloys. Electrochem. Solid-State Lett. 3, 543–545.

[B45] Younes O. Gileadi E. (2002). Electroplating of Ni/W Alloys. J. Electrochem. Soc. 149, C100–C111. 10.1149/1.1433750

[B46] Younes-Metzler O. Zhu L. Gileadi E. (2003). The Anomalous Codeposition of Tungsten in the Presence of Nickel. Electrochimica Acta 48, 2551–2562. 10.1016/s0013-4686(03)00297-4

[B47] Zhang Y. Bilan H. K. Podlaha E. (2018). Enhancing the Hydrogen Evolution Reaction with Ni-W-TiO2 Composites. Electrochemistry Commun. 96, 108–112. 10.1016/j.elecom.2018.10.015

[B48] Zhang Y. Podlaha-Murphy E. J. (2017). Electrodeposition of Ni-Fe-Mo-W Alloys – Parts 15. Prod. Finishing 81, 9–16.

[B49] Zou Y. Xiao Z. Fei X. Ren X. (2004). Preparation Technology and Properties of Nickel-Tungsten-Titanium Dioxide Composite Plating. Mater. Prot. 37, 24–26.

